# Design and Synthesis of Multi-Functional Superparamagnetic Core-Gold Shell Nanoparticles Coated with Chitosan and Folate for Targeted Antitumor Therapy

**DOI:** 10.3390/nano11010032

**Published:** 2020-12-24

**Authors:** Sharafaldin Al-Musawi, Salim Albukhaty, Hassan Al-Karagoly, Faizah Almalki

**Affiliations:** 1 Faculty of Biotechnology, Al-Qasim Green University, Babylon 51013, Iraq; 2 Department of Chemistry, College of Science, University of Misan, Maysan 62001, Iraq; albukhaty.salim@uomisan.edu.iq; 3 Department of Internal and Preventive Medicine, Veterinary Medicine College, University of Al-Qadisiyah, Al-Diwaniyah 58002, Iraq; hassan.aliwee@qu.edu.iq; 4 Faculty of Science, Taif University, Taif 21944, Saudi Arabia; fa.ahmad@tu.edu.sa

**Keywords:** superparamagnetic iron oxide nanoparticles, gold nanoparticles, doxorubicin, chitosan, breast cancer, folate

## Abstract

A dual-targeting nanomedicine composed of pH-sensitive superparamagnetic iron oxide core-gold shell SPION@Au, chitosan (CS), and folate (FA) was developed as a doxorubicin (DOX) antitumor medication. Microemulsion was used for preparation and cross-linking conjugation. The characteristics of the designed nanocomposite were studied using atomic force microscopy (AFM), transmission electron microscopy (TEM), scanning electron microscopy (SEM), X-ray diffraction, UV-visible spectroscopy, Zeta potential and vibrating sample magnetometry (VSM), and Fourier transform infrared spectroscopy. The prepared SPION@Au-CS-DOX-FA nanoparticles (NPs) were spherical with an average diameter of 102.6 ± 7 nm and displayed an elevated drug loading behavior and sustained drug release capacity. The SPION@Au-CS-DOX-FA NPs revealed long term anti-cancer efficacy due to their cytotoxic effect and apoptotic inducing efficiency in SkBr3 cell lines. Additionally, Real-time PCR outcomes significantly showed an increase in BAK and BAX expression and a decrease in BCL-XL and BCL-2. In vivo results revealed that SPION@Au significantly decreased the tumor size in treated mice through magnetization. In conclusion, prepared SPION@Au-CS-DOX-FA could be a beneficial drug formulation for clinical breast cancer treatment.

## 1. Introduction

Breast cancer is a major recurring cause of death for women worldwide; hence, the development of modern diagnostic methods for breast cancer in the early stages is essential for the possible treatment of this disease [1,2]. Conventional treatment methods generally include surgery, chemotherapy, and radiotherapy to eradicate tumors. Most chemotherapy agents have a systemic effect, are insufficiently targeted, and poorly penetrate the intended treatment site [3]. Doxorubicin (DOX) is one of the most efficient anti-cancer substances and is extensively used to treat breast cancer and other malignancies such as bladder, ovarian, and lung cancer [4,5]. However, its clinical use is limited due to its dose-dependent cell toxicity (myelosuppression and cardiotoxicity), multidrug resistance development, and low specificity against cancer cells [6,7]. Current research progress on nanotechnology has led to the development of nanocarrier-drug delivery vehicles and their potential applications in gene delivery and cancer therapy [8,9,10]. Superparamagnetic nanoparticles (NPs) (SPIONs) have emerged as a superior agent in tumor therapy because of their unique properties, particularly the superior magnetism that permits non-invasive magnetic resonance imaging (MRI) and in vivo applications such as cancer tissue hyperthermia by a targeted technique in the presence of an external magnetic field [11,12,13,14,15]. This field promotes the cellular uptake of NPs containing magnetic materials. The combination of Au NPs and SPION enables drug accumulation in a specific location in the body by using a magnetic field for effective targeted therapy at the tumor site [16]. The Au-coated SPION is a robust platform and a suitable agent for improving stability, biocompatibility, and surface interaction [17]. The chitosan (CS) surface is a natural cationic polysaccharide that exhibits low toxicity, biocompatibility, and hydrophilicity and is subsequently considered for DOX delivery [18]. This surface has been widely used for the dispersion of SPIONs in an aqueous solution. Folate (FA) receptor is highly overexpressed on many surfaces of cancer cell lines [19]. FA-coated SPION NPs exhibit significant cellular uptake improvement compared with unmodified NPs [20]. This study aimed to develop a multifunctional nanoparticle composed of biocompatible factors (SPION, Au, CS, and FA) and analyze its ability to reduce DOX toxicity and its applicability for targeted cancer cell therapy. In addition, the therapeutic effects were assessed on in vitro and in vivo animal model tumor cancer cells.

## 2. Materials and Methods

### 2.1. Chemical Materials

Ferric chloride, hexahydrate (FeCl_3_·6H_2_O ≥ 99%), and ferrous chloride tetrahydrate (FeCl_2_·4H_2_O ≥ 99%), strong Ammonium hydroxide solution (99%) 25% (*v*/*v*), 3-(4,5-dimethyl-2-thiazolyl)-2,5-diphenyl-2H-tetrazolium bromide (MTT), and dimethyl sulfoxide (DMSO) (≥99.8%) were purchased from Merck (Merck, Germany). SkBr3 and MCF 10A cell lines were acquired from ATCC (Manassa, CO, USA). DMEM medium and other factors were obtained from Gibco (Gibco, UK).

### 2.2. Preparation of SPIONs

SPIONs were prepared and optimized through chemical co-precipitation [21].

#### Synthesis of SPION@Au Core-Shell and SPION@Au-CS-DOX-FA NPs

SPION@Au was fabricated following a previous procedure [22] with slight modifications. In brief, 12.5 g of SPIONs were dispersed in a beaker with a flat bottom containing 250 mL of distilled water and 0.1 M HAuCl_4_ (≥99.9%) ·4H_2_O mixture for 15 min. The mixture was ultrasonicated a beaker with a flat bottom for 15 min, and incubated at 38 °C for easy Au3+ adsorption into SPION surface. The excess gold ions are typically extracted by centrifugation for 30 min at (10,000 RCF). Then, the clear supernatants were included in practical use. Glucose (1 mg/5 mL) was used as a reducing factor in the mixture, which was shaken and incubated at room temperature. The formed SPION@Au composite was then cleaned with distilled water several times until the pH scale reached 7 and was analyzed by atomic force microscopy (AFM, OMCL-RC800PSA-1, Olympus, Tokyo, Japan), scanning electron microscopy (SEM, Carl Zeiss, Supra 55, Oberkochen, Germany), transmission electron microscopy (TEM, Carl Zeiss, Gottingen, Germany), UV-visible spectroscopy (Shimadzu, Tokyo, Japan) and X-ray diffraction XRD (SIEMENS-D5000, Karlsruhe, Germany). For synthesizing of SPION@Au-CS-DOX-FA, SPION@Au was coated with CS and FA using a previous method with some modification [23]. In brief, 250 mg of CS (Mw = 50–190 kDa) was added to 17.5 mL of acetic acid (1%, *v*/*v*) under stirring for 15 min at room temperature until a homogeneous solution was obtained. The prepared solution was then applied to the SPION@Au NPs. Afterward, 50 mg of DOX (1 mg/mL) drug was dissolved in 25 mL of distilled water and added to the SPION@Au-CS-DOX-FA solution. FA (25 mg) was previously dissolved in (5 mL) distilled water. Approximately 50 μL of NaOH solution (10 M) was mixed under magnetic stirring, and 0.5 mL of the prepared FA was obtained. The solution was mixed with 5 mL (1 mg/mL) of SPION@Au-CS-DOX-FA NPs. The solution was incubated at room temperature in a shaking incubator (300 rpm) for two h to facilitate DOX uptake in SPION@Au-CS-FA NPs. Optical density (OD) for DOX residual in the supernatant was measured using a UV-vis spectrometer device on 498 nm.

### 2.3. Size and Morphological Characteristics of Prepared NPs

Zeta potential analyzer (Malvern Instruments Ltd., Malvern, UK) was used to detect the NPs average size diameter, polydispersity index (PDI), and zeta potentials. SPION@Au-CS-DOX-FA was also analyzed by AFM (OMCL-RC800PSA-1, Olympus, Tokyo, Japan) at room temperature using a drop of fresh solution on silicon that was left to dry. SEM was characterized after sputter coating with Au for samples, and TEM at 200 kV accelerating voltage was employed to observe the morphological characteristics of SPION@Au-CS-DOX-FA. The samples were prepared by sticking a drop of dispersed particle suspension on the carbon-coated copper grid and allowing it to dry at room temperature. Furthermore, the prepared SPION@Au-CS-DOX-FA was evaluated by Fourier transform infrared spectra (FTIR, Shimadzu Corporation, Tokyo, Japan) in the range of 4000–400 cm^−1^. A vibrating sample magnetometer (VSM) was used to perform magnetic tests at room temperature.

### 2.4. Determination of Release Profile and DOX Calibration Curve

The DOX drug released from loaded SPION@Au-CS-FA NPs was studied under in vitro conditions using phosphate buffer (0.01 M, pH 7.4) and citrate buffer (0.01 M, pH 5.4) containing 2 M sodium salicylate in a shaker incubator at 37 °C. In brief, 1 mL of nanoparticle solution was placed in a dialyze bag placed in 100 mL of citrate or phosphate buffer. Tween 80 was selected as a surfactant agent to emulsify and inhibit the sedimentation volume from a released drug. UV-vis measurement was used to measure the DOX loading efficiency. The following Equation (1) can measure the loading content (LC %) and loading efficiency (LE %) of DOX:

(1) $$Encapsulation\;Efficiency\;\left(\%\right)=\frac{\left(Total\;amount\;of\;drug\;-\;Free\;amount\;of\;drug\right)}{Total\;amount\;of\;drug}\times 100$$

### 2.5. Cell Culture

SkBr3 and MCF-10A cell lines were cultured in 100 mL culture flasks using Dulbecco’s modified Eagle medium (DMEM) cell culture medium, 10% phosphate-buffered saline (PBS), and 1% penicillin-streptomycin (1 × 10^4^ units per mL). The culture was incubated at 37 °C in a humidified atmosphere containing 5% CO_2_.

#### Cellular Internalization

SPION@Au-CS-DOX-FA was functionalized with fluorescein 5(6)-Isothiocyanate (FITC) to evaluate its cell internalization efficiency using a fluorescence microscope (Nikon Eclipse TE2000-U, Temecula, CA, USA). The fluorescein isothiocyanate (FITC)-chitosan was synthesized by coupling fluorescein isothiocyanate (5-isomer) to chitosan polymer through a reaction between FITC’s isothiocyanate group and the primary chitosan amino group. For the analysis of cellular internalization, FITC-SPION@Au-CS-DOX-FA was examined in SkBr3 and MCF-10A cell lines through fluorescent emission from DOX in cell medium. The cells were seeded with DMEM medium on coverslips in six-well plates to allow attachment overnight. The medium was detached and washed by (PBS) The cells were treated with FITC-SPION@Au-CS-DOX-FA for 4 h at 37 °C. After incubation, the medium was removed, and the cells were washed with PBS three times. Coverslips were placed on a microscope slide. The cells were treated with 5 g of FITC-SPION@Au-CS-DOX-FA for 3 h. Subsequently, the nanocomposite-containing medium was discarded, and the cells were washed with PBS. 

### 2.6. MTT Assay

The potential cell toxicity of SPION@Au-CS-DOX-FA was examined using MTT assay. SkBr3 and MCF-10A cells were seeded into 96-well plates at a density of 5 × 10^4^ cells per well and incubated for 24 h in DMEM culture medium, which was then removed and replaced by a new one with different concentrations of SPION@Au-CS-DOX-FA suspension. DOX concentrations ranging from 4 μg/mL to 20 μg/mL were added to the wells. The cell cultures were incubated at 37 °C for 24 h, and 20 μL of MTT (5 mg/mL in PBS pH 7.4) was added to each well and incubated for 4 h. The upper-medium from wells was gently removed, and 150 μL of DMSO was added. The suspension was vigorously mixed, transferred into microtubes, and centrifuged at 13,000 rpm for 10 min. Absorbance was read using a microplate reader (BioTek Power Wave XS, VT, USA) at 570 nm.

### 2.7. Flow Cytometry Test

Annexin V-FITC kit (Beyotime, Biotechnology Co., Ltd., Nantong, China) was used to calculate the percentage of cells rapidly undergoing apoptosis by flow cytometry in accordance with the manufacturer’s instructions. The culture plated of SkBr3 and MCF-10A were resuspended in binding buffer. The cells at a density of 10^3^ cells/well were combined with 5 µL of Annexin V-FITC and 5 µL of PI. At room temperature, the cells were then incubated in the dark for 15 min. Flow cytometry was conducted using the FACSCalibur™ system (BD Biosciences, Franklin Lakes, NJ, USA).

### 2.8. Apoptosis Detection by Quantitative Real-Time PCR

#### 2.8.1. Total Isolation of RNA and Complementary DNA Synthesis

cDNA (Fermentas, Hanover, Germany) was synthesized directly from extracted total RNA through reverse transcription following the instruction protocol.

#### 2.8.2. Quantitative Real-Time PCR Reaction

Primers for target and endogenous genes (Bcl-2, Bcl-xl, Βak, and BAX) were designed using software primer express (Table 1). PCR primers (forward and reverse sequences) were obtained from previous studies [24,25,26,27], and β-actin was employed as a housekeeping gene for normalizing the cDNA variation. The gene expression was studied using RT-PCR (Applied Biosystems, Foster City, CA, USA).

### 2.9. In Vivo Study

#### 2.9.1. Animal Use

NOD.CB17-Prkdcscid/J mice 6–8 weeks old and weighing 25 g were acquired from the animal house. The guideline approved animal care and use of Animal Care and Research Committee of Al-Qasim Green University (ethics committee approval code: 533FD2) was adopted from the guideline for the care and use of laboratory animals. All in vivo protocols and methods were performed by pertinent guidelines and regulations. Moreover, all experimental procedures were confirmed by an Animal Care and Research Committee of Al-Qasim Green University.

#### 2.9.2. Tumor Volume and Survival Rate Studies

A total of 1 × 10^6^ SkBr3 exponentially growing cells/200 μL of PBS-free medium were injected subcutaneously (s.c.) in NOD.CB17-Prkdcscid/J mice. Tumor mass was confirmed on day 8 due to the fast growth and offensive characteristics of the SkBr3 cell line. The animals were divided randomly into four groups (n = 7 per group), including three test groups intravenously receiving 12.5 mg/kg body weight DOX loaded NPs and free DOX for 3 weeks and three control groups including SPION@Au-CS-DOX-FA and PBS.

Tumor volume (mm^3^) was calculated three times per week using a digital Vernier caliper (Mitutoyo, Japan) using the Equation (2).

*Tumor volume* = ½ [*Tumor length* × (*Tumor width*)^2^] (2)

### 2.10. Splenocyte Proliferation Index

The mice were sacrificed, and a part of the spleen from each animal was isolated. Splenocyte proliferation index was calculated using the method of Babaei et al. [28]. The spleens were removed, cleaned with PBS, homogenized, and passed through a 100 μM filter to acquire a single-cell suspension. The cells were centrifuged, and the trypan blue exclusion test was used to determine viable splenocytes. The tumor cell lysate was used as a positive control, and the medium served as a negative control. MTT assay was used to determine the splenocyte proliferation index. Each experiment was accomplished in triplicate wells.

### 2.11. Cytokine Detection by ELISA

The splenocytes were incubated with tumor lysate to test their effects on the cytokine release. Phytohemagglutinin (PHA) was added as a positive control, and the medium was used as a negative control. ELISA kit (R&D, Minneapolis, MN, USA) was used to collect and test the supernatant for Interferon-Gamma (IFN-γ) and Interleukin 4 (IL-4.)

### 2.12. Statistical Analysis

Statistics analysis was conducted by one-way ANOVA and unpaired students t-test by using Tukey’s multiple comparisons. Significance level was set at * *p* < 0.05; ** *p* < 0.01, and *** *p* < 0.001;

## 3. Results and Discussion

A multilayered nanocomposite named SPION@Au platform was prepared through microemulsion, stabilized by biocompatible CS, and conjugated with targeting FA. DOX chemotherapeutic drug was loaded, and their synergistic action for tumor therapy was evaluated. Chemical co-precipitation was adopted to generate SPIONs because it allows for feasible large-scale and relatively low-cost production [29]. Au shell protects SPION against oxidation and makes it sensitive to light absorption [30]. CS was used as a stabilizing factor in the presence of FA for nanoparticle surface modification to prevent SPION agglomeration [31]. DOX-loaded FA-CS-SPION@Au NPs had a size particle average of 102.6 ± 7 nm. Encapsulation was performed under the electrostatic load capacity of DOX to SPION@Au-CS-FA NPs by using the electrostatic interaction between the carboxyl group of carboxylic acid-functional iron-oxide NPs and the DOX amine group [32].

### 3.1. Characterization of SPION@Au-CS-DOX-FA

**Figure 1:**
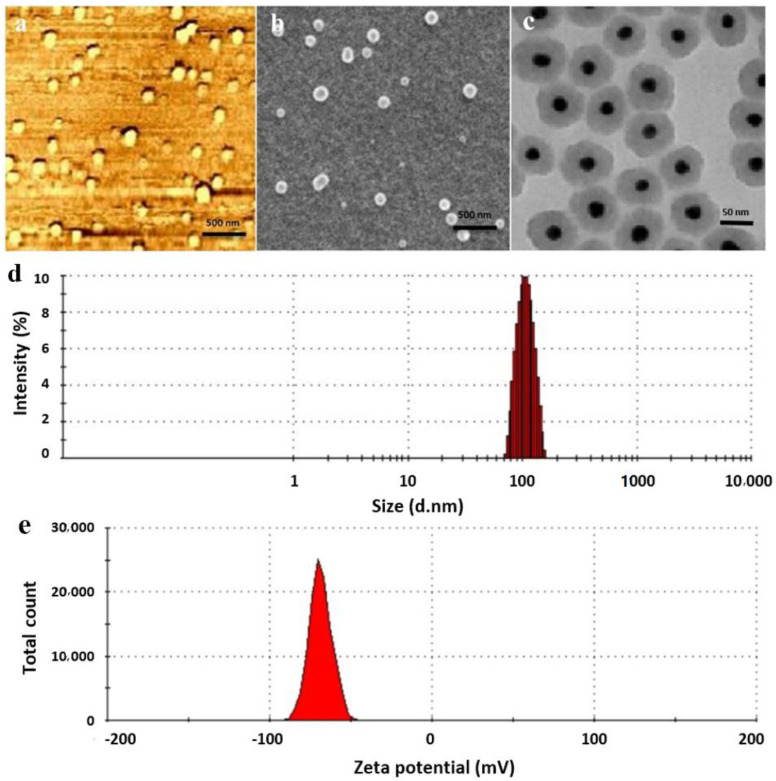
Characterization of the designed (SPION@Au-CS-FA nanocomposite) with different techniques. (**a**) atomic force microscope (AFM), (**b**) scanning electron microscope (SEM), (**c**) transmission electron microscope (TEM) images, (**d**) the particle size distribution of SPION@Au-CS-FA and (**e**) charge measurement uses dynamic light scattering (DLS). Abbreviations: FA: Folate, CS: Chitosan, SPION: Superparamagnetic iron oxide nanoparticles (NPs), and Au: Gold.

**Figure 2:**
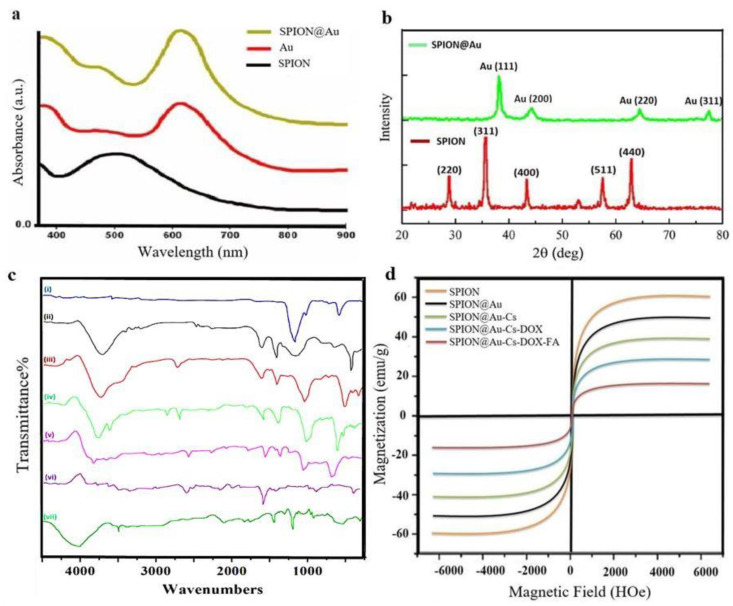
UV visible (**a**) and XRD (**b**) characterization of the prepared SPION@Au core-shell NPs. FT-IR spectra (**c**) of the prepared SPION@Au-CS-doxorubicin (DOX)-FA NPs. Au (ⅰ), SPION (ii), SPION@Au-Cs (iii) SPION@Au-CS-DOX-FA (iv), and DOX (v), FA (vi) Cs (vii).magnetic hysteresis curve (**d**) of the prepared SPION@Au-CS-DOX-FA NPs.

The prepared SPION@Au-CS-DOX-FA core-shell NPs had a spherical shape and uniform size as observed by AFM, TEM, and SEM (Figure 1a–c). An average diameter of 102.6 ± 7 nm with single peak shape and narrow particle size distribution (Figure 1d) with zeta potential −63.1 ± 6 mV in the neutral environment of pH 7.4 (Figure 1e). Additionally, the changes in the surface plasmon resonance (SPR) band were found in the SPION, and SPION@Au spectra as shown by UV visible spectroscopy (Figure 2a). The absorption of pure SPION decreased with the wavelength of light. The large SPR bands with a wavelength peak range from 550 nm to 650 nm often indicate Au shell’s presence on the surface of SPION. Figure 2b displays the powder X-ray diffraction (XRD) patterns of the SPION and SPION@Au core-shell NPs. The diffraction angle of the raw SPION (311) peak occurs at 39.51°, which means that the SPION composition is magnetite before the Au shell is reduced. After encapsulating SPION with Au, the XRD signals shift to (111) indicated the Au shell formation on the surface of SPION. The XRD signals of the Fe_3_O_4_ core were shielded by the gold layer because of the heavy atom effect. Alteration in the chemical structures of NPs resulted in differences in dynamic light scattering (DLS) and zeta potential values (Table 2).

**Figure 3:**
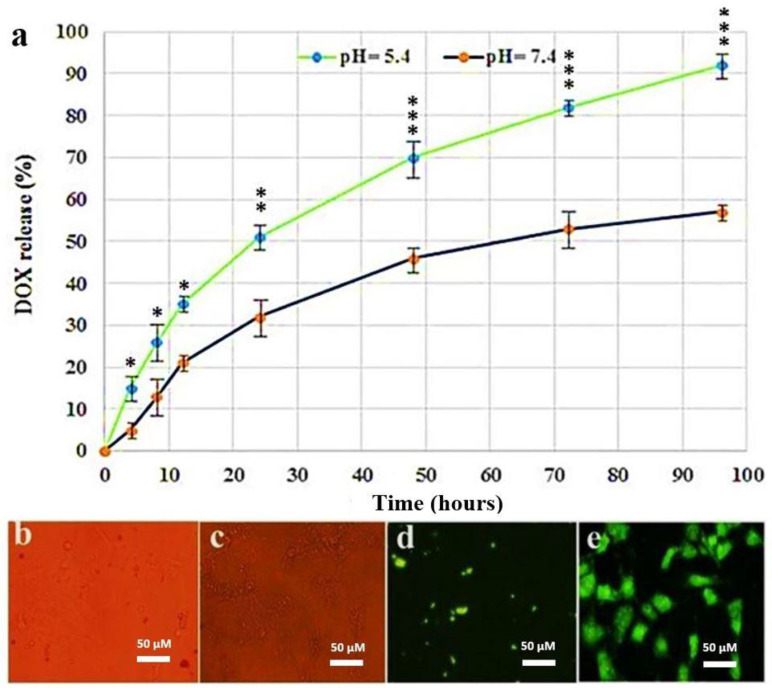
In vitro release profile of DOX from SPION@Au-CS-FA NPs at different pH values and the intracellular uptake. (**a**) the release rate of SPION@Au-CS-DOX-FA NPs at pH 7.4 and pH 5.4. All experiments were performed at 37 °C. The data represent mean ± S.D., (* *p* < 0.05; ** *p* < 0.01; *** *p* < 0.001); n = 3). (**b**) optical microscopy image of SPION@Au-CS-DOX-FA treated cells. (**c**) Optical microscopy image of free DOX. (**d**) fluorescence microscopy image of SPION@Au-CS-DOX-FA treated cells. (**e**) fluorescence microscopy image of free DOX.

The nanoscale is important because nanocarrier systems containing anti-cancer drugs can easily target and accumulate within the tumor’s area by exerting cytotoxic effects on proliferating cells [33]. FTIR confirmed that the prepared NPs contained uncoated SPION, SPION@Au, FA, and CS NPs. The spectra for SPION, Au, CS, DOX, and FA are illustrated in (Figure 2a). Absorption peaks were found at (443, 583, and 634 cm^−1^) and can be related to the Fe–O bond [34]. The band at 3455.8 cm^−1^ was related to the stretching vibration. O-H group in citrate capped SPION@Au range, and its intensity was lower than that in the uncoated Fe_3_O_4_ because of Au coating. CH2 stretching allows the average intensity up to 3016.7 cm^−1^ [35]. CS FTIR spectrum showed a band that corresponded to the O-H stretch at ~3484 cm^−1^. The amide group in CS was evident from its peak of absorption at ~1660 cm^−1^ [36]. In addition, the FTIR FA spectrum showed IR bands at 1480–1700 cm^−1^ corresponding to C=N bonds, C=O, and the NH_2_-pteridine ring group in folate [37]. VSM was used to study the magnetic activity of the SPION@Au-CS-FA NPs (Figure 2b). This finding revealed the superparamagnetic properties of SPION@Au-CS-FA NPs with a saturation magnetization value of 51.2 emu/gm. The rapid response to the external magnetic field, the stable formulation, and the desired stability of SPIONs in solution make these particles suitable nanocarriers [38]. The capacity of SPION@Au-CS-FA NPs for DOX release was studied under different pH conditions of 5.4 and 7.4 at 37 °C in PBS [39]. The amounts of (DOX) drug released from FA-CS-SPION@Au NPs were detected by calculating the supernatant’s fluorescent emission intensity at various pH values (Figure 3a). The pH of the culture medium was found vital for the release capacity of DOX from FA-CS-SPION@Au NPs. The pH values for evaluating drug release were selected based on the physiological and endosomal pH value of cancer cells. Drug release curves showed that the release time from loaded SPION@Au-CS-DOX-FA within 96 h was slower when treated with a phosphate buffer in normal pH (pH 7.4) compared with that under acidic citrate buffer a pH value of (pH 5.4). By contrast, the in vitro release profiles of free DOX indicated an analogous release algorithm in (pH 7.4 and 5.4). These curve results revealed a faster DOX release rate in pH 5.4 at similar conditions compared with that in pH 7.4.

### 3.2. Cellular Uptake Ability

The release amount of DOX drug-loaded SPION@Au-CS-DOX-FA NPs was internalized into the cytoplasm of the SkBr3 cell line, as shown in (Figure 3b–e) respectively. This is shown by the incremental enhancement of the DOX loaded green fluorescence SPION@Au-CS-DOX-FA NPs from 0 to 6 h. Besides, green fluorescence has been shown to emanate only from the internalized NPs.

### 3.3. MTT Assay

**Figure 4:**
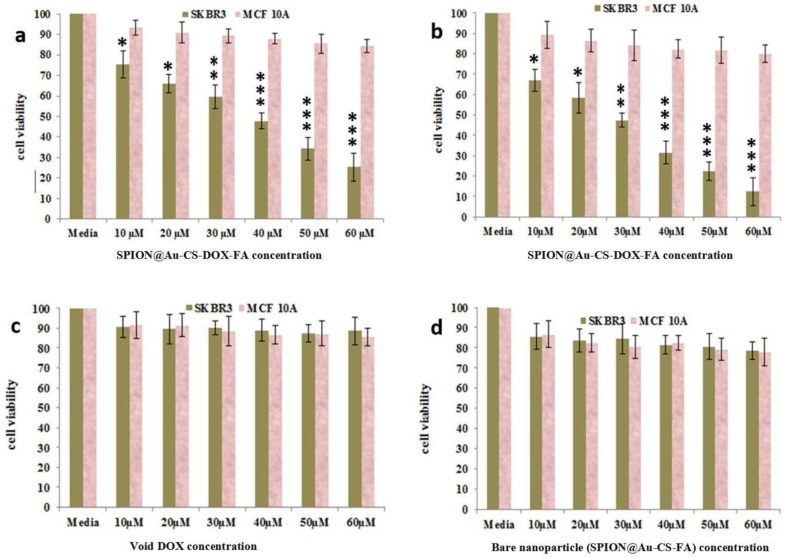
Cell toxicity evaluation of SPION@Au-CS-DOX-FA nanoformulation by the MTT assay on SKBR3 and MCF-10A cell lines with 24 h (**a**), 48 h (**b**) incubations. The cells also treated with void DOX (**c**) & bare SPION@Au-CS-FA nanoparticle (**d**) with same concentrations for 48 h. (mean ± S.D., (* *p* < 0.05; ** *p* < 0.01; *** *p* < 0.001); n = 3).

The in vitro cell toxicity of SPION@Au-CS-DOX-FA was examined by MTT assay against SKBR3 and MCF-10A cell lines (Figure 4). The cancer cells were treated with free DOX and bare SPION@Au-CS-FA. Even at the highest concentration of 60 μM, no toxicity for cells was observed, and more than 85% of cells still survived after 48 h of incubation, which indicated cytocompatibility. Besides, SPION@Au-CS-DOX-FA significantly decreased cell viability and induced higher inhibition activity of cancer cells compared with free DOX and bare SPION@Au-CS-DOX-FA NPs alone. IC_50_ concentration was determined by a dose-response curve fitting of the cell viability date. The IC_50_ values for SPION@Au-CS-DOX-FA NPs for SK BR3 cancer cell line within 24 and 48 h were 29.11 and 14.68 µM, respectively. This phenomenon occurred because apoptosis was more intense than necrosis after the treatment with SPION@Au-CS-DOX-FA NPs.

Furthermore, the same concentrations of SkBr3 cancer cells did not affect the proliferation of MCF-10A cells even at 60 μM, indicating the safe use of these NPs. The use of NPs as capsules for anti-cancer drugs can facilitate their intake by the cells and lysosomes, leading to a highly induced cytotoxic activity [40]. One previous investigation indicated that SPIONs dose-dependently increases toxicity [41]. However, elucidating the mechanism underlying the anti-proliferative potentials of SPION@Au-CS-DOX-FA is essential for standardizing and developing an efficient treatment regime.

### 3.4. Flow Cytometry

**Figure 5:**
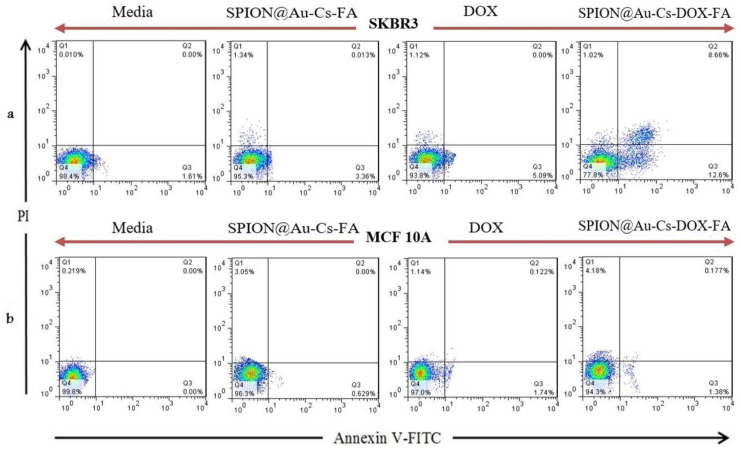
Apoptosis induction of SkBR3 (**a**) and MCF-10A (**b**) cell lines with media (control), bare SPION@Au-CS-FA NPs, free DOX and SPION@Au-CS-DOX-FA. The number of SkBR3 cells undergoing apoptosis significantly increases when treated with SPION@Au-CS-DOX-FA. Treatment of SkBr3 cell line with bare SPION@Au-CS-FA and free DOX separately, indicating that both treatments did not show any remarkable apoptosis induction.

The apoptosis of SkBr3 and MCF-10A cell line cultures was analyzed using flow cytometry. For the cell death onset analysis, SkBr3 (Figure 5a and MCF-10A (Figure 5b) cell lines were handled for 48 h separately using SPION@Au-CS-FA, DOX, and SPION@Au-CS-DOX-FA. Apoptotic cell death was analyzed and studied by flow cytometry by staining the cells with Annexin-V-FITC. The results showed a dose-dependent induction in these two cell lines of early or late death of apoptotic cells as present in (Figure 5). Compared with MCF-10A cells, SK-BR3 cells exhibited more cell death with a percentage of 22.4% apoptosis following the administration of SPION@Au-CS-DOX-FA (5 μM; *p* < 0.05). Meanwhile, 6.4% apoptosis was observed in MCF-10A cells under the same conditions (*p* < 0.05), suggesting that SPION@Au-CS-DOX-FA could greatly increase the DOX-induced apoptosis in breast cancer cells. Annexin V-FITC binds with a high affinity ideally to phosphatidylserine and therefore, can be used to determine the degree of apoptosis [42]. The number of cells in the early and/or late stage of apoptosis was quantified with PI staining. The results suggest that SPION@Au-CS-DOX-FA increases the DOX sensitivity of SkBr3 cells and significantly induces apoptosis in cancer cells (Figure 5a).

### 3.5. Real-Time PCR and Gene Expression

**Figure 6:**
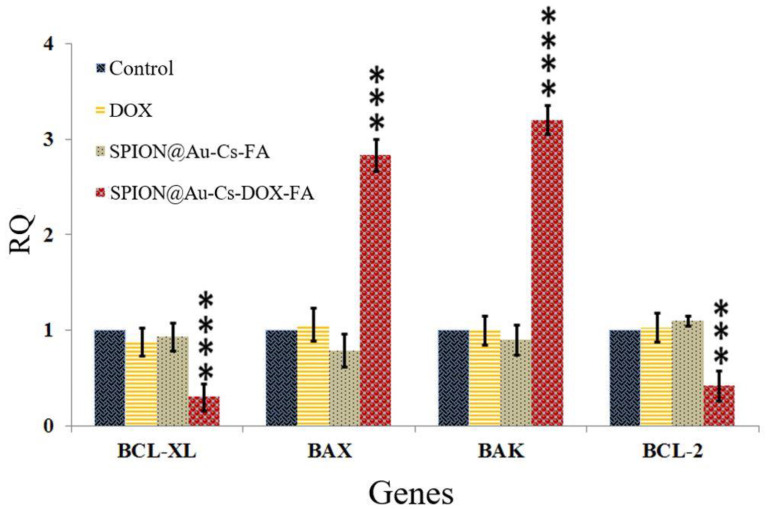
Gene expression of BCL-XL, BAX, BAK, and BCL-2 have been analyzed in four groups of SkBr3 cells. In each group, the first column represents control cells, the second column for cells treated with DOX, the third column for cells treated with SPION@Au-CS-FA, and the fourth column for cells treated with SPION@Au-CS-DOX-FA. The values in the graph indicate the mean ± SD. *** *p* < 0.001, **** *p* < 0.0001 represent significant differences between the control (untreated) and other treatments.

After treatment with FA-CS-DOX-SPION-Au, the expression level of the selected genes was compared between SkBr3 cell lines treated with SPION@Au-CS-FA and DOX individually. The statistical study of real-time PCR revealed that after treatment, FA-CS-DOX-SPION-Au significantly decreased the expression of Bcl-2 and Bcl-xl by 0.32-fold (*p* < 0.001) and 0.18-fold (*p* < 0.0001), respectively, compared with those of the control group (untreated). Moreover, FA-CS-DOX-SPION-Au considerably (*p* < 0.0001) up-regulated the expression of BAK by 2.97 and Bax by 2.58-fold of normal levels (*p* < 0.01, Figure 6). In general, B-cell lymphoma 2 (BCL-2) is a family of receptor proteins that regulate apoptosis via proteins pro-apoptotic (Bax, Bad, and Bak) and anti-apoptotic (BCL-2 proper, BCL-XL, and MCL), anti-apoptotic protein overexpression leads to treatment resistance [43]. These findings indicated that DOX anti-cancer drug-loaded FA-CS-SPION-Au NPs induce apoptosis by regulating the family Bcl-2 proteins.

### 3.6. Effect of SPION@Au-CS-DOX-FA on Tumor Volume and Survival Rate

**Figure 7:**
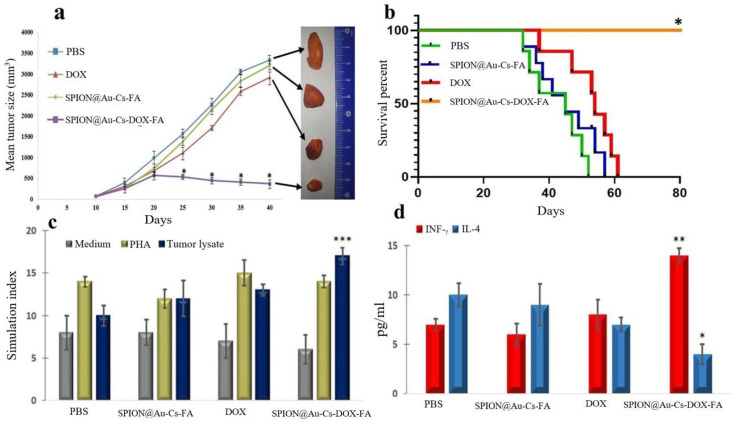
The in vivo antitumor effects of SPION@Au-CS-DOX-FA in a mice tumor model. Changes in the tumor size in representational mice (four groups). (**a**,**b**) on day 19 the tumor growth in mice treated with SPION@Au-CS-DOX-FA was reduced significantly (*p* < 0.01) relative to that of the other groups received free DOX, SPION@Au-CS-FA NPs, and PBS. (**c**) the proliferation index of splenocytes following the induction of tumor lysate, PHA (positive control), and medium (negative control). (**d**) splenocytes collected from mice treated by SPION@Au-CS-DOX-FA were re-stimulated with tumor lysate. A substantial increase (*p* < 0.05) in IFN-ÿ levels compared with those of other groups and a significant decrease (*p* < 0.05) in IL-4 production was observed in the mice receiving SPION@Au-CS-DOX-FA nanoformulation compared with those given DOX, SPION@Au-CS-FA, and PBS. Results indicate the mean of measurements carried out in triplicate (mean ± S.D., (* *p* < 0.05; ** *p* < 0.01; *** *p* < 0.001); n = 3).

The tumor volume changes in representational mice (four groups) are shown in Figure 7a. On day 19, the tumor growth in mice treated with SPION@Au-CS-DOX-FA was reduced significantly (*p* < 0.01) relative to that of the other groups receiving free DOX, SPION@Au-CS-FA NPs, and PBS. Small particles in the tumor can pass easily through the capillary wall but can also be pushed out of the tumor by blood flow. Given that the magnetic force acting on the magnetic particles is proportional to the particle volume, the fluidic drag force may overcome the magnetic force experienced by the small particles [44].

### 3.7. Evaluation of the Lymphocyte Proliferation Index Following Treatment

All four groups of mice were examined for the proliferation index of splenocytes following the induction of tumor lysate, PHA (positive control), and medium (negative control). Significantly, high levels of splenocyte proliferation were found in the mice received the nanoformulations of SPION@Au-CS-DOX-FA compared with those of three other classes (*p* < 0.05, Figure 7c). By contrast, the splenocytes from the other three groups showed no significant differences.

### 3.8. Estimation of IFN-γ and IL-4 Level Post-Treatment

Figure 7c,d suggested that the splenocytes collected from mice treated with SPION@Au-CS-DOX-FA were re-stimulated with tumor lysate and showed a substantial increase (*p* < 0.05) in IFN-γ levels compared with the other groups. However, the average level in all samples was slightly small. A significant decrease (*p* < 0.05) in IL-4 production was observed in the mice received SPION@Au-CS-DOX-FA nanoformulation compared with those taken DOX, SPION@Au-CS-FA, and PBS. Stimulated sample results for the PHA (positive control) and medium (negative control) showed no significant differences between the groups. SPION@Au-CS-DOX-FA NPs showed antitumor properties and influenced the immune system. The proliferative index of splenocytes and secretion of IFN-γ and IL-4 cytokines were also investigated. Such findings are in agreement with other previous studies suggesting a strong association between the antitumor effect of DOX-loaded polymeric nanocarrier and immune reactions, including splenocytes proliferation and cytokine production [45,46]. In addition, changes in cytokine development, such as antitumor response are correlated with IFN-γ [47].

## 4. Conclusions

The SPION@Au-CS-DOX-FA NPs showed new activity with the release of the pH-dependent drug. These NPs act as a platform for DOX drug magnetic vehicle with low cytotoxic effects. Furthermore, in vivo data showed that the tumor development was significantly (*p* < 0.01) inhibited in the mice treated with SPION@Au-CS-DOX-FA NPs compared with those treated with free DOX. We thus conclude that SPION@Au-CS-DOX-FA could be developed into a novel formulation which showed remarkable improvement in synergistic therapeutic effects of magnetic drug nanocarriers through their binding with anti-cancer drug DOX for use in clinical treatment of cancer in the future.

## Figures and Tables

**Table 1:** Sequences of primers oligonucleotides used to amplify the studied genes.

Gene	Forward Primer Sequence	Reverse Primer Sequence	Ref.
**β-actin**	5′-CTGGCACCCAGCACAATG-3′	5′-GCCGATCCACACGGAGTACT-3′	[24]
**Bcl-2**	5′-TGCCTTTGTGGAACTGTACG-3′	5′-GGCCAAACTGAGCAGAGTC-3′	[25]
**BAX**	5′-AGCTGCAGAGGATGATTGC-3′	5′-GTTGAAGTTGCCGTCAGAAA-3′	[25]
**Bcl-xl**	5′-AAGGAGATGCAGGTATTGGTGAGT-3′	5′-CCAAGGCTCTAGGTGGTCATTC-3′	[26]
**Βak**	5′-ACTGGGATCGAGACATGTG-3′	5′-AGAAGGTGATGTGTACATTGC-3′	[27]

**Table 2:** Hydrodynamic scale and zeta potential parameters of the synthesized NPs.

	SPION	SPION@Au	SPION@Au-CS	SPION@Au-CS-FA	SPION@Au-CS-DOX-FA
**Size**	23.1 ± 2	37.3 ± 3	68.3 ± 6	81.8 ± 3	102.6 ± 7
**Zeta**	−35.2 ± 2	−43.4 ± 7	50.2 ± 3	24.8 ± 4	−63.1 ± 6
